# The regulative role and mechanism of BNST in anxiety disorder

**DOI:** 10.3389/fpsyt.2024.1437476

**Published:** 2024-12-04

**Authors:** Mingjun Xie, Ying Xiong, Haijun Wang

**Affiliations:** School of Traditional Chinese Medicine, Shandong University of Traditional Chinese Medicine, Jinan, China

**Keywords:** anxiety, neural circuit, bed nucleus striatum, regulation, multimodal techniques

## Abstract

Anxiety disorders, common yet impactful emotional disturbances, significantly affect physical and mental health globally. Many neuron circuits are associated with anxiety regulation like septo-hippocampal loop, amygdala(AMYG), bed nucleus of the stria terminalis (BNST), ventral hippocampus (vHPC), and brain regions like medial prefrontal cortex (mPFC). However, the concrete mechanism of anxiety disorder in BNST is relatively unknown. Recent research showed BNST plays a critical role in modulating anxiety owing to its anatomical location and special circuit characteristics, which are considered to be a hub in the limbic system regulating anxiety. BNST consists with multiple subregions, which can project separately into different brain regions and exert projecting independently to various brain regions with distinct regulatory effects. Moreover, multiple signal pathways in BNST are reported to play significant roles in regulating anxiety and stress behavior. This review briefly describes anxiety disorders and subdivisions and functions of BNST, focusing on the main neural circuits that serve as fundamental pathways in both the genesis and potential treatment of anxiety disorders and the molecular mechanism of BNST on anxiety. The complexity of structures and mechanisms has facilitated the development of imaging techniques. Innovative multimodal imaging techniques, such as functional magnetic resonance imaging (fMRI) and positron emission tomography (PET), have non-invasively illuminated BNST activities and their functional connections with other brain areas. These methodologies provide a deeper understanding of how BNST responds to anxiety-inducing stimuli, offering invaluable insights into its complex role in anxiety regulation. The continued exploration of BNST in anxiety research promises not only to elucidate fundamental neurobiological mechanisms but also to foster advancements in clinical treatments for anxiety disorders.

## 1 Introduction

Anxiety is a normal emotional expression that signifies heightened arousal and negative emotions (1) that can enhance alertness even when there is no immediate threat (2). It can potentially increase one’s awareness and aid in survival by enabling quick reactions to potential danger. This emotional state can be elicited by stimuli that do not pose immediate harm or arise internally. On the other hand, fear is associated with responding to an actual or perceived imminent threat and diminishes as the threat subsides (2). While occasional anxiety is common in healthy individuals, persistent, disruptive, or disproportionate anxiety in the face of real dangers can result in a constant state of excessive tension and fear, indicating is a pathological condition. Pathological anxiety is classified into three main categories by the Diagnostic and Statistical Manual of Mental Disorders, fifth edition: obsessive-compulsive and related disorders, trauma- and stressor-related disorders, and anxiety disorders (3). Excessive anxiety, termed an “anxiety disorder”, is a maladaptive mood disorder characterized by persistent worry, despair, tension, and distress, along with physical symptoms like tachycardia, nervousness, and difficulty relaxing (4, 5).

**Figure 1:**
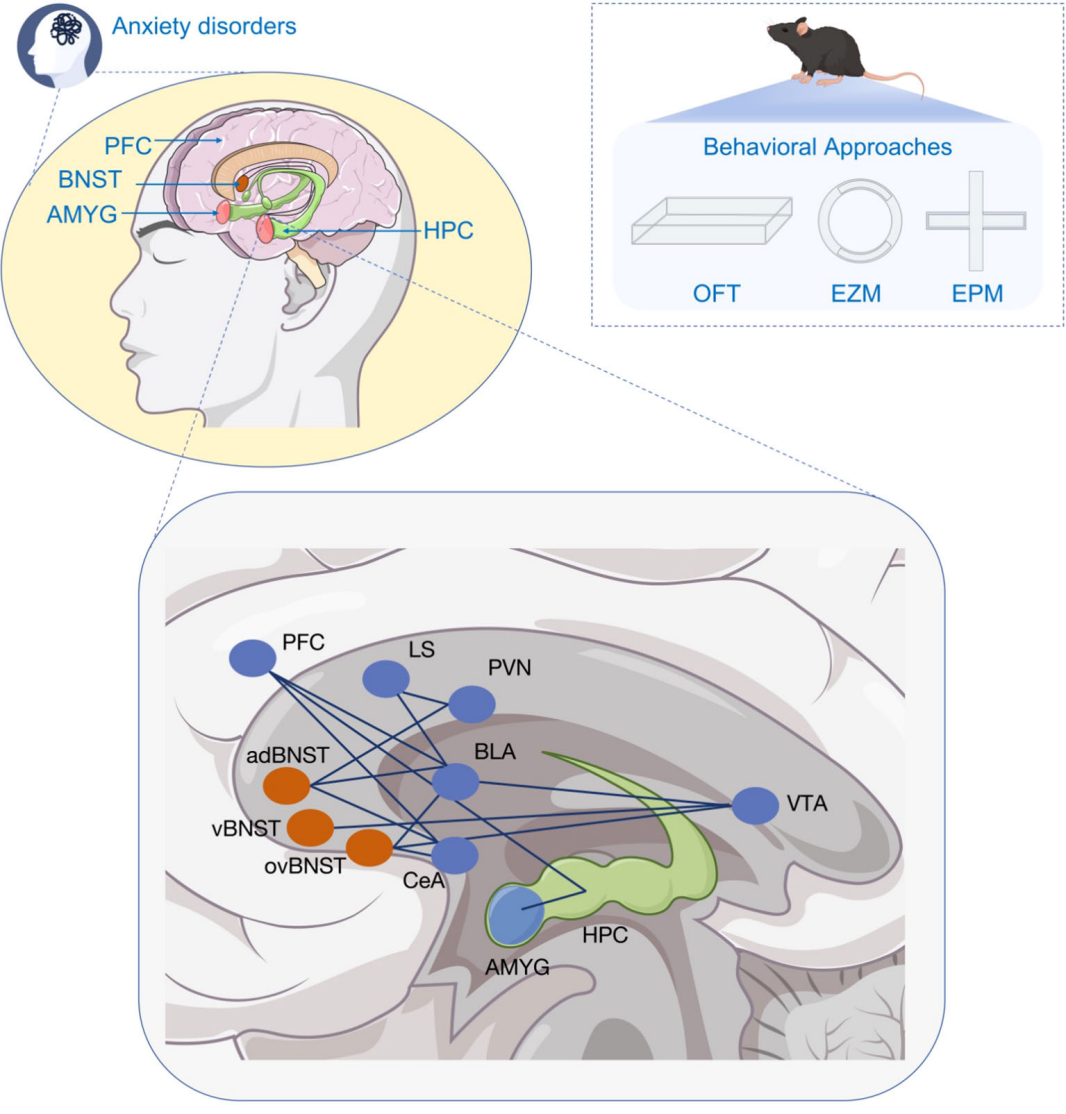
Brain regions and neural circuits associated with anxiety disorders.

There are some brain regions and neural circuits related with anxiety disorders (**Figure 1**). Among them, the bed nucleus of the stria terminalis (BNST) is an essential stress-responsive region that regulates anxiety response due to its location and circuit characteristics (6). Multiple signal pathways in BNST are reported to play significant roles in regulating anxiety and stress behavior. Li et al. found that histamine receptors expressed in BNST neurons and infusion of histamine into the BNST exerted an anxiogenic effect. In contrast, the blockade of histamine receptors reduced the anxiogenic effect induced by acute restraint stress without influencing behaviors in normal rats (7). It is found that increasing the expression of SIRT1 in male mouse BNST could ameliorate anxiety behaviors induced by chronic stress (8). In addition, H1 and H2 receptors are also discovered to be involved in regulating anxiolytic effect in stressed rats (7). Similarly, CB1 and CB2 receptors controlled anxiety-like behaviors and they were located in the anterior and posterior divisions of the BNST (9). There are also sexual differences in the protein expression and subsequent behavior in BNST. Rigney et al. discovered that downregulation of AVP regulation in BNST reduced aggressive behavior without influencing anxiety-like behavior in males. While for females, none of the behaviors were altered (10). However, human females are prone to having anxiety-related disorders. More research is needed to be explored to solve the physiological basis with the help of high-end equipment.

In anxiety research, integrating multimodal techniques has greatly enhanced the investigation of bed nucleus striatum (BNST) function over the years. These techniques encompass a diverse array of methodologies, including neuroimaging, optogenetics, chemogenetics, electrophysiology, and molecular genetics (11). By combining these complementary approaches, researchers are able to dissect BNST circuits with spatial and temporal resolution, allowing for the interrogation of neural activity, connectivity, and molecular signaling pathways involved in anxiety regulation. The use of these advanced multimodal techniques offers unprecedented opportunities to explore BNST neurobiology with high precision and sensitivity, providing insights into its involvement in anxiety regulation. We delve into the significance of these techniques in BNST anxiety research, emphasizing their contributions to elucidating BNST function at both macroscopic and molecular levels.

Important brain regions related to anxiety disorders include BNST,PFC, AMYC, and HPC, along with the main neural circuits that function as crucial pathways in both the development and potential therapies for anxiety disorders. These roles can be assessed through behavioral testing methods in animals. adBNST Anterodorsal bed nucleus of the stria terminalis, AMYC Amygdala, BLA Basolateral Amygdala, BNST Bed nucleus of the stria terminalis, CeA central amygdala, EZM Elevated zero maze, EPM Elevated plus maze, HPC Hippocampus, LS lateral septum, OFT open field test, ovBNST Ovoid bed nucleus of the stria terminalis, PFC Prefrontal cortical, PVN paraventricular nucleus, vBNST Ventral bed nucleus of the stria terminalis, VTA ventral tegmental area.

## 2 Anxiety disorders

### 2.1 General

Anxiety disorders are a common emotional disorder that seriously affects people’s physical and mental health. Anxiety disorders are one of the more common mood disorders, significantly impacting individuals’ overall well-being and mental health and placing a substantial burden on society. Studies have indicated that the onset of anxiety disorders can occur as early as 11 years old, with the prevalence among adults reaching 18% and a lifetime prevalence rate of 28.8%. In Western countries, anxiety disorders are the most prevalent neuropsychiatric disorders (12, 13) with a recent 3-year multi-method study across 30 European countries and a population of 514 million people revealing that anxiety has the highest 12-month prognosis among psychiatric disorders at 14% (14). As a result of their high prevalence, chronic nature, and comorbidity, anxiety disorders are ranked as the ninth leading cause of damage to health by the World Health Organization (14).

Anxiety is primarily characterized by persistent arousal, alertness, fear, concomitant defenses, and autonomic response changes (82). The main anxiety disorders include separation anxiety disorder and selective mutism, specific phobias, social anxiety disorder, generalized anxiety disorder, panic disorder, and agoraphobia (15).

### 2.2 Primary behavioral tests for anxiety disorders

To study the pathological mechanisms of anxiety disorders effectively, anxiety-related behavioral tests in experiments must meet specific criteria. Currently, researchers utilize various methods to assess anxiety by observing externally visible phenotypes, which are crucial for drug development studies focusing on stress and anxiety modeling (16). Among various model organisms, rodents are preferred for investigating the neural circuit mechanism of anxiety disorders. In “approach-avoidance” conflict tasks, mice with avoidance-like anxiety traits tend to remain in the enclosed safe area of the behavioral device. The more commonly used behavioral paradigms for anxiety assessment include the elevated plus maze (EPM), zero maze (EZM), and open field test (OFT). In the EPM behavioral paradigm, anxious animals avoid the open arm and favor the closed arm of the maze (17). In the zero-maze, anxious animals tend to stay within the closed quadrant (18). In the OFT, organisms display anxious attributes by predominantly moving along the perimeter of the maze. Researchers often assess anxiety-like behaviors through the analysis of social interactions among rodents (19). For instance, young mice emit ultrasound vocalizations at fear-associated frequencies when separated from their breeder’s cage, the pups emit ultrasounds at frequencies associated with fear and anxiety responses, which can be reduced effectively by anxiolytic drugs (20). Alongside behavioral experiments, stress hormone levels and vital signs are often monitored as indicators of anxiety in rodents. These physiological indicators are based on the manifestations of anxiety in clinical settings, such as increased sweating, dizziness, elevated heart rate, and blood pressure (21).

## 3 Important neural circuits that regulate anxiety

### 3.1 General

In the early stages of anxiety research, researchers focused on identifying the brain nuclei associated with anxiety by observing behavioral changes in experimental organisms following the destruction of specific nuclei. The type of nuclei associated with anxiety, such as the amygdala(AMYG), BNST, ventral hippocampus (vHPC), and brain regions like medial prefrontal cortex (mPFC), were visually determined through these methods. However, there has been limited exploration into the internal loops of brain regions regulating anxiety. Modern techniques such as optogenetics have enabled researchers to delve deeper into anxiety research by selectively manipulating neural circuits. By controlling the projections of specific neuron types towards downstream brain regions, researchers can gain valuable insights into the relationship between anxiety-like behaviors and their neural circuits. Anxiety is influenced by both local or long-range connections among various brain regions, including AMYG, HPC, BNST, and PFC (19). Patients with generalized anxiety disorder exhibit dysfunctions primarily in the PFC (22), while individuals with obsessive-compulsive disorder (OCD) show changes in the structure and function of the striatum (Corpus striatum, CS) and thalamus (Thalamus, Thal). These interconnected brain regions form anxiety neural circuits involving basolateral amygdaloid nucleus (BLA)- vHPC, mPFC-vHPC, and BNST-central amygdala (CeA) pathways. The AMYG serves as the central hub for information processing, interacting with the vHPC through the BLA. Activation of the BLA-vHPC synapse promotes anxiety, while its inhibition reduces anxiety levels. Additionally, the BNST and CeA, collectively known as the expanded amygdala, regulate anxiety through dense projection (23) The mPFC also contributes to fear and anxiety regulation, particularly innate anxiety, by cooperating with the vHPC.

### 3.2 Modulation of anxiety by other neural circuits

The septo-hippocampal loop plays a crucial role in detecting conflict and uncertainty in anxious environments, thereby enhancing arousal and attention in organisms (24). This loop involves the lateral septum (LS), a component of the septal system that is closely related to the regulation of stress-induced anxiety (25). Pharmacological inactivation of one side of the ventral hippocampus and the contralateral LS hemisphere can reduce anxiety levels in organisms by disrupting the connection between them (26). In addition, manipulating the projections of CRHR2-expressing GABAergic neurons from the LS to the anterior hypothalamus (AH) can heighten anxiety levels (27). Activation of these GABAergic neurons suppresses neural activity in the AH. Interactions among these brain regions trigger enduring neuroendocrine and behavioral aspects of anxiety (28).

Clinical data indicate that alterations in the volume of the amygdala, such as an increase (29) or a decrease (30), are associated with anxiety disorders. Notably, increased AMYG anxiety is observed in individuals exhibiting symptoms of social anxiety disorders (31). Animal studies have demonstrated that the destruction of the AMYG is a key factor in anxiety disorders (32). These findings underscore the significant role of AMYG activity in anxiety disorders and suggest the presence of functionally distinct subregions within the AMYG that interact with other brain regions to regulate anxiety. BLA contains basolateral and basomedial subregions and receives excitatory inputs from the thalamus and sensory cortex (33). The BLA processes this input information and establishes a link between external stimuli and emotional responses, evaluating different threats and rewards (34). Anxiety levels are modulated by various mechanisms in the absence of threat presence (35). Optogenetic activation of specific neuron types in the BLA region increases anxiety levels, while stimulating excitatory inputs from the BLA to the central amygdala (CeL) produces anxiolytic effects (36), suggesting that different downstream projections from the same region may have contrasting behavioral effects. Moreover, direct stimulation of granule cells in the ventral dentate gyrus (DG) alleviates anxiety-like behaviors in mice (37).

The brain area responsible for evaluating threatening signals in the mPFC, which first processes threatening stimulus signals before eliciting a subcortical response. The mPFC receives inputs from various regions such as the thalamus, amygdala, and hippocampus, and projects to areas like the amygdala and striatum (38). Studies have demonstrated that inhibiting mPFC-CeA projections can dampen neuronal activity in CeA brain regions, and lead to anxious behavior. Moreover, environmental threat stimuli influence the interconnection between the mPFC and the BLA brain region, when BLA neurons are activated, the mPFC rhythmically discharges and transmits safety signals to alleviate anxiety levels (39). Neurons within the mPFC can encode anxiety-related features in different environments (40, 41). However, the precise role of the neural circuitry associated with projections from the ventral hippocampus to the mPFC in anxiety regulation remains unclear. Further investigations using optogenetic-specific manipulation of these projections to downstream loci (IL or PL) to assess their impact on anxiety are necessary. The prefrontal cortex plays a dynamic role in modulating organism anxiety levels in response to varying degrees of threatening stimuli (42).

### 3.3 Modulation by the basolateral amygdala-terminal bed nucleus output loop

The BNST processes fear and perseverative response afferents, which are directly innervated by BLA afferents, as well as inputs from the HPC and mPFC, Glu from the entorhinal cortex, and the insular cortex (43). The presence of redundancy in the functions of the AMYG and BNST ensures that fear learning remains robust even in the face of localized damage and dysfunction. For instance, if the BLA function is absent, the BNST compensates by playing a role in fear memory acquisition, albeit requiring more training for BLA-independent fear learning (44). In addition, specific subregions within the BNST have been found to selectively modulate different features of the anxiety phenotype. BLA inputs to the anterior ventral BNST (anterodorsal BNST, adBNST) facilitate behavioral and physiological anxiolysis; while localized inhibition of a BNST subregion, the nucleus of the ovoid nucleus (ovBNST), to the adBNST induces anxiety. These findings further emphasize the significant role of the BNST in anxiety regulation (21).

## 4 Bed nucleus of stria terminalis

### 4.1 Current status of research on bed nucleus of stria terminalis

The BNST, located in the medial caudate nucleus, is a complex limbic forebrain region that handles stress and reward, housing various subregions and specific neuronal cell populations (45). Functioning as a pivotal control hub for motivation and emotional states, the BNST plays an important role in perceiving threatening stimuli (80), orchestrating a neural network that interacts with multiple brain regions to process a wide array of external signals and modulate behavioral responses (46). For example, neural pathways linking the BNST to the hypothalamus foster eating and drinking behaviors while maintaining internal homeostasis through connections with the brainstem (47). Additionally, connections between the BNST and the LS and medial amygdala (MeA) regulate reproductive behavior across species (48). Together with the central AMYG and the nucleus ambiguus (NAc), the BNST forms the extended amygdala (49). Functional magnetic resonance studies have revealed significant activation of BNST neurons in patients with anxiety disorders, with clinical evidence suggesting its involvement in the modulation of persistent fear and anxiety, as well as regulating aversive and reward-related behaviors. With its extensive interconnections with various brain regions and diverse receptor subgroups, the BNST has emerged as a pivotal region in the investigation of mood-related psychiatric disorders, establishing itself as a key player in the anxiety-related brain circuitry.

### 4.2 Structural subdivisions and main functions of the BNST

It is essential to understand the organization of the BNST brain region and its subregions to explore the mechanisms of integrating information and executive functions (80, 50). Located in the ventral septal nucleus, the BNST is situated near the ventral septal nucleus, the anterior region of the hypothalamus, and fluctuates within and down in the anterior commissure region (49). The BNST can be subdivided into the anterior BNST (aBNST) and posterior BNST (pBNST), with aBNST primarily regulating specific emotions and pBNST mainly involved in reproductive behavior.

The aBNST consists of several major subregions, including the ovoid nucleus (ovBNST), anterolateral BNST (alBNST), anterodorsal nucleus (adBNST), saccular nucleus (juxtacapsulae BNST, juBNST) (fusiform BNST, fuBNST), rhomboid BNST (rhBNST), ventral BNST (vBNST), and dorsomedial BNST (dmBNST), each of these subregions can project separately to different brain regions and exert projecting independently to various brain regions with distinct regulatory effects (51).

The ovBNST plays a crucial role in integrating information related to negative emotional stimuli through neurotransmitters such as corticotropin-releasing hormone (CRH), GABA, dopamine (DA), and dynorphin, thereby influencing anxiety-like behavior (21). It forms strong connections with other brain regions, transmitting GABAergic projections to the CeA and receiving inputs from dopamine receptors in the VTA and the DR (52). Increased CRH and mRNA expression in the ovBNST follow certain chronic restraint stress and plantar electric shock (53).

In the alBNST, various receptors like oxytocin, dopamine, and serotonin influence functional regulation, with GABAergic nerve fiber transmitting output to the PVN of the hypothalamus (PVN). Neuronal subpopulations expressing these receptors integrate neural inputs, regulate mood and pain, and receive Glu inputs from the VTB and PVNventral hypothalamic peduncle (26).

The vBNST region contains dense noradrenergic fibers (54), receiving cytosolic inputs from the caudal ventral medulla and the nucleus tractus solitarii (52), enabling the inhibition of cardiovascular responses and facilitation of norepinephrine release in response to stimuli, which is also facilitated by the administration of plantar electric shock stimulation. The vBNST ultimately facilitates sensitive responses to facial stimuli, leading to adaptive behaviors.

### 4.3 Advances in research on anxiety disorders in BNST

Numerous experimental pharmacological studies have demonstrated the significant role of BNST in modulating anxiety-related behaviors (55). Patients exhibiting anxiety symptoms exhibit elevated neuronal activity in the BNST region in response to uncertain stimuli, in contrast to the normal population (56). Clinical imaging studies have further revealed a positive correlation between BNST activity and the severity of anxiety symptoms (81). Boucher et al. found that the activation of pituitary adenylate cyclase-activating polypeptide (PACAP) receptor and the lateral parabrachial nucleus (LPBn) played an important role in producing anxiety-like behavior (57). In addition, researchers illustrated how chronic pain induced anxiety behavior via regulating BNST. The molecular genetic approach verified a specific subpopulation of BNST neurons expressing cocaine- and amphetamine-regulated transcript (CART) was elevated by chronic pain, which further led to increased inhibitory inputs to LH-projecting BNST neurons. Finally, the anxiety-like behavior was induced (58). Histamine receptors were also reported to induce anxiety behavior. By blocking histamine H1 or H2 receptors in BNST, the anxiety behavior was reduced (59). Based on those experiments, the regulation mechanism of BNST on anxiety behavior is complicated and variable. Owing to the complexity of the BNST structure and involved underlying mechanism, integrating multimodal techniques encompassing neuroimaging, optogenetics, chemogenetics are applied to investigating molecular mechanisms.

It has been illustrated through optogenetic manipulation studies that different subregions of the BNST and their projections to distinct downstream neural circuits can trigger varying anxiety-like behaviors. The ovBNST and adBNST, as two subregions of the BNST, exhibit opposing roles in anxiety regulation. Manipulation of three separate downstream neural circuits from adBNST to the LH, VTA, and parabrachial nucleus (PBN) resulted in mice displaying anxiety-related behaviors and diverse functional phenotypes (21). Studies have also demonstrated that the vBNST in the anterior part of the BNST transmits excitatory Glu and inhibitory GABAergic projections to non-dopaminergic neurons in the VTA. Optogenetics activation of Glu neurons in the vBNST projecting to the VTA induces avoidance behaviors and increases anxiety levels, while activation of the GABAergic neurons in the vBNST to the VTA promotes rewarding and anxiolytic behavior (60). The relationship between cellular and behavioral functions is evidenced in the enhanced activity of Glu neurons and the suppressed activity of GABA neurons in the vBNST following aversive plantar electrical stimulation (61). This highlights the distinct roles played by different types of BNST neuronal projections to the VTA in anxiety production. Understanding the specific neuron types within the BNST and their downstream projections to the neural circuits is crucial for unraveling the mechanisms underlying anxiety regulation (19). On the other hand, the results of functional MRI scans showed that compared with normal controls, generalized anxiety disorder patients exerted increased activity in the BNST (56). BNST is thought to be involved in more chronic regulation of sustained anxiety. Whereas, the dynamics of activation in this region are rarely known, and a novel image technique is needed to solve this problem.

## 5 Modal imaging techniques in exploring anxiety in BNST

Recent advancements in neuroscience research have led to significant breakthroughs in the study of anxiety disorders and other mood disorders. Particularly, researchers have shown great interest in investigating the role of the BNST brain region as a key player in the development of anxiety disorders. Traditional imaging techniques had limitations in accurately depicting the link between BNST and anxiety due to its small size. However, the development and utilization of multimodal imaging techniques have enabled researchers to overcome these limitations and more precisely uncover the connection between BNST and anxiety. These studies not only enhance our comprehension of anxiety disorder pathogenesis but also lay a crucial groundwork for future clinical diagnosis and treatment.

One notable advancement in neuroscience and psychology is the progress within fMRI technology. fMRI has significantly enhanced spatial and temporal resolutions, leading to advancement in data analysis methods and the exploration of brain functional connectivity. Through task activation experiments, resting-state functional connectivity experiments, and brain network analysis, researchers have delved deeper into the functional connectivity among different brain regions (62, 63). This exploration has helped us understand the relationship between these connections and various aspects such as cognitive functions, emotion regulation, and diseases, thereby enhancing our insight into the brain’s functional structure and dynamic regulatory mechanisms. Examining recent studies in this field clarifies the significance and utility of multimodal imaging techniques in unraveling the neural mechanisms of anxiety disorders. These studies have immense potential to clinical practice positively by providing new insights for diagnosis and treatment strategies.

### 5.1 Neural control of optogenetic and tracer technologies in BNST and anxiety

The BNST serves as a crucial emotion regulation center, playing a significant role in the processing of anxiety-related signaling input and output pathways. Integrated incoming information from brain regions such as the amygdala and prefrontal cortex occurs within the BNST, which subsequently transmits signals through output pathways connected to brain regions like the hypothalamus. Disruptions in this signaling pathway can result in an anxiety regulation imbalance, potentially contributing to the development and onset of anxiety disorders. Various studies have demonstrated the therapeutic potential of interventions targeting the BNST neural circuitry. For example, manipulating the activity of BNST neurons through optogenetic techniques or pharmacological agents has shown promise in mitigating symptoms associated with anxiety disorders, thereby introducing novel avenues for their treatment (64).

The complexity and significance of BNST neural circuits in the etiology of anxiety disorders underscore the need for further research efforts. Continued investigation will support a comprehensive understanding of the pathophysiological mechanisms underlying anxiety disorders, offering a theoretical framework and clinical insight for the refinement of more effective therapeutic interventions. Tracer and optogenetic techniques represent valuable tools for probing these neural circuits. Tracer technology facilitates the delineation of intricate neural connections between the BNST and other brain regions by labeling and tracing neuronal trajectories and connections (65). Conversely, optogenetic techniques enable precise control over neuron activity (66), thereby elucidating their specific contributions to anxiety regulation. The integration of these methodologies enhances our ability to gain a nuanced understanding of the role played by neural circuits in the pathogenesis of anxiety disorders, fostering fresh perspectives and strategies for the management of such conditions.

#### 5.1.1 The BNST and the amygdala in anxiety

The amygdala and the BNST both play important roles in emotion regulation, with the amygdala being more focused on rapid, localized fear processing and fear memory, while the BNST is involved in the long-term evaluation of threats and emotion regulation. They form a complex network of emotion regulation. The amygdala is recognized for its role in fear memory, particularly in relation to the processing of threatening stimuli and fear mechanisms. In contrast, the BNST is more associated with enduring anxiety mechanisms, specifically the long-term assessment and emotional regulation of potential threats in uncertain situations. The BNST regulates emotional responses and stress levels through connections with structures such as the amygdala and hippocampus, influencing the pathogenesis of anxiety disorders by contributing to the persistence and recurrence of anxiety.

Russell et al. (67) suggests that neurons in the anterior subdivision of the BA basal amygdala region (aBA) without projections to the dorsal BNST (dBNST) are implicated in contextual fear engrams, whereas neurons projecting to the dBNST do not seem to partake in contextual fear engrams directly. Instead, they may serve as a pathway from the BA to the ovBNST, activated in the initial encoding of contextual fear memories. Neurons projecting to the dBNST showed activity during the initial encoding phase of situational fear memories, indicating their importance in emotional learning. However, these neurons are not directly involved in the long-term storage of situational fear memories. The ovBNST, a subregion of BNST, receives the unidirectional transmission from BA neurons, suggesting that amygdala-BNST projections play a crucial role in the processing of situational fear memories, which may contribute to anxiety onset and maintenance by transforming and integrating fear memories. The BNST is highlighted as a crucial component in anxiety regulation, acting as a key relay station in the brain’s emotion-emotion regulation loop. Neural projections from the basal amygdala via the BA to ovBNST pathway are believed to be involved in the initial encoding of situational fear memories. BNST projections to the central nucleus modulate fear responses to unlabeled threats and cued fear. The complex neurotransmission between BNST and different amygdala subregions may enable BNST to integrate information, ultimately influencing behavioral manifestations of anxiety.

Increased BNST activity, influenced by inhibitory influences from the centromedian amygdala (CM), could result in heightened anxiety states through hyperactivation of downstream targets involved in autonomic, neuroendocrine, and/or behavioral regulation (68).

#### 5.1.2 Interaction of the BNST with neural circuits

Through the application of Restraint Stress modeling in conjunction with optogenetic, chemogenetic, and neural tracer techniques, Luchsinger (69) found that activation neural projections from the INSULAR to the BNST heightened anxiety-like behaviors, while inhibiting this circuit resulted in anxiolytic effects. Another study (70) found that neurons in the paraventricular thalamus (PVT) receive excitatory neural inputs from glutamatergic neurons in the insula cortex and send outputs to glutamatergic neurons in the BNST, forming a loop that modulates anxiety behavior induced by restraint stress in mice. This study strategically manipulated the activities of insular cortex (IC), PVT, and BNST neurons using optogenetic and chemical genetic techniques, uncovering their interconnections and their roles in modulating anxiety behaviors. Specifically, in response to stress, the IC activates the PVT, which further activates the BNST, ultimately influencing susceptibility to anxious behavior. The identification of this novel neural loop paves the way for an enhanced understanding of anxiety disorders. Furthermore, Xiao et al. (71) observed that sNAcPV neurons in a mouse model of chronic stress displayed heightened excitability, leading to increased avoidance behavior. This also unveiled new GABAergic neural pathways from adBNST to sNAcPV neurons, and new GABAergic neural pathways originating from the anterior dorsal amygdala (adBNST) to sNAcPV neurons. Optogenetic activation of GABAergic neurons in adBNST was found to decrease the excitability of sNAcPV neurons, resulting in anxiolytic effects. Additionally, it was noted that the majority of GABAergic input neurons express growth inhibitory hormone (SOM). The coordination of SOM- and PV-cell functions in the BNST to NAc circuit was found to exert an inhibitory effect on anxiety-like responses, highlighting the intricate neural mechanisms involved in anxiety regulation.

### 5.2 Neuroplasticity of the BNST in anxiety regulation

In a mouse model, chronic social defeat (CSD) stress has been demonstrated to induce mitochondrial dysfunction within the BLA, triggering the activation of the PINK1-Parkin-dependent mitochondrial autophagy pathway. This perturbation resulted in an upsurge in mitochondrial autophagy, causing an excessive elimination of mitochondria from the BLA. Consequently, neurotransmission from the BLA to the BNST was disrupted. This particular pathway is considered pivotal in modulating anxiety levels in the brain. The study effectively showcased that employing optogenetic techniques to boost synaptic transmission from the BLA to the BNST could successfully ameliorate anxiety behaviors induced by CSD stress. This discovery highlights the potential therapeutic efficacy of targeting the BNST in anxiety management. The optogenetic activation not only enhanced the synaptic function of the BLA-BNST pathway but also led to a significant reduction in anxiety-like behaviors in mice, thereby establishing a direct link between BNST neuroplasticity and anxiety regulation.

### 5.3 Anxiety modulation in the BNST at the molecular level

Previous studies have explored the relationship between the BNST molecule and anxiety **Table 1**, focusing on the role of CRH, a hormone produced by the supraoptic nucleus of the hypothalamus. CRH regulates cortisol release by activating the hypothalamic-pituitary-adrenal (HPA) axis, which is central to the stress response. Additionally, CRF expression in the BNST has been associated with stress and anxiety behaviors. The BNST is a key region of CRF expression (6) and various subnuclei within the BNST show heightened levels of CRF in rats, mice, and rhesus monkeys. This increased CRF expression in the BNST may contribute to the development of chronic stress and anxiety disorders. For example, chronic stress can alter synaptic plasticity in CRF-expressing neurons, potentially leading to long-term potentiation (LTP) and the manifestation of chronic stress and anxiety symptoms (72). Moreover, The FK506 binding protein51(FKBP51), an auxiliary protein of heat shock protein 90 kDa (Hsp90) encoded by the Fkbp5 gene, is a recognized risk factor for anxiety-related disorders and stress dysregulation. A study manipulating FKBP51 expression in the ovBNST demonstrated anxiolytic effects when FKBP51 was overexpressed and increased anxiety phenotypes upon FKBP51 knockout. This highlights the importance of FKBP51 expression and regulation in the ovBNST for normal anxiety-related behaviors (73). In a recent investigation (8) reduced SIRT1 expression in the BNST and corticotropin-releasing factor (CRF) expression were observed in mice model anxiety induced by chronic stress exposure. Local overexpression of activated SIRT1 in the ovBNST reversed anxiety behaviors in these mice, decreased CRF upregulation, and normalized the overactive CRF neurons. The mechanism of action involved SIRT1 enhancing GR-mediated CRF expression by interacting with the Glucocorticoid Receptor (GR) co-chaperone FK506 Binding Protein 5 (FKBP5), leading to enhanced CRF transcriptional repression. Current results are oriented from basic experiments like mouse or rat anxiety models, while there are rarely reports about clinical data or targeted medicine. The associated molecule or signal pathway is multiple and their signal axis action site is also variable, which may lead to difficulty in targeting. Hence, more clinical data about anxiety in BNST is needed to investigate.

**Table 1:** Summarization of recent effective molecule on anxiety in BNST.

Molecule	Expression	Effect	Reference
CRF	Upregulated	Promoting the development of chronic stress and anxiety disorders	(72)
FKBP51	Downregulated	Increasing anxiety phenotypes	(73)
SIRT1	Downregulated	Decreasing CRF expression and over-activating CRF neurons.	(8)
5-HT	Upregulated	Enhancing fear and anxiety and activating a subpopulation of corticotropin-releasing factor (CRF)	(55)
Pituitary adenylate cyclase-activating polypeptide (PACAP)	Upregulated	Modulating BNST function and increasing anxiety-like behavior.	(57)
Ketamine	Upregulated	Ketamine-induced anxiety-like behaviors	(78)
Haloperidol (HAL) and aripiprazole (ARI)	Upregulated	Suppressing CRH expression in BNST	(79)

Furthermore, Wang et al. (74) have identified different cellular subpopulations in the adBNST, such as corticotropin-releasing hormone-positive (CRH+) and protein kinase C-d-positive (PKC-d+) neurons, which exhibit varied emotional behavior. CRH+ and PKC-d+ neurons receive inputs from similar brain regions, and exhibit significant variations in their downstream projection density, thereby providing new perspectives on the circuit organization of adBNST neurons (75). Furthermore, the study reveals that chronic social isolation (PWSI) stress triggers androgynous-specific anxiety-like behavior by enhancing the excitability of DRD2+ neurons within the dorsal striatum bed nucleus (dBNST). These neurons serve as a crucial neural mechanism underlying PWSI-induced sex-specific behavioral abnormalities and may represent a potential therapeutic target for addressing social stress-related mood disorders (75). Additionally, Assis et al. (76) demonstrates that, in stressful situations, the endogenous cannabinoid (eCB) system in the BNST is activated to counterbalance the effects of stress. This signaling pathway within the BNST appears to play a pivotal role in modulating anxiety-like behaviors, particularly in individuals with prior stress exposure. Finally, Li et al. (7) observed that histaminergic neurons in the hypothalamus have a direct projection to the BNST. Blocking or downregulating histamine H1 and H2 receptors in the BNST attenuates anxiety triggered by acute restraint stress, indicating that histamine signaling in the BNST is crucial for modulating anxiety behaviors. Therefore, inhibition of histamine receptors could be a promising therapeutic approach for treating anxiety disorders. Garcia et al. (77) found that under natural conditions, 5-Hydroxytryptamine (5-HT) release in the dBNST modulates anxiety-like behavior through 5-HT1A receptors. The activation of 5-HT input to the dBNST decreases anxiety. These findings suggest a complex role for 5-HT in regulating dBNST function. These studies suggest that the regulation of BNST on anxiety is not only influenced by the projection of hypothalamic neurons, but also differs in the composition of multiple subregions and the regulation by different hormones. Therefore, the study on the structural complexity of BNST is of great significance for revealing the pathological mechanism.

## 6 Conclusion and prospect

This article mainly introduces the molecular mechanisms of anxiety regulation of anxiety in BNST. The BNST is a sexually dimorphic structure and at present, the effect of gender difference on the regulation of BNST on anxiety is still controversial and needs further study. The subregions and their distinct reactivity all deserve further investigation, which might provide insight into a better understanding of the mechanism of anxiety in BNST. By combining techniques such as optogenetics and fMRI, we can more precisely study the association between the activity patterns of BNST neural circuits and anxiety behaviors. In the future, we anticipate an increase in studies utilizing neuroengineering tools like brain-computer interface technology and neuromodulation devices to explore the association between the BNST and anxiety in more dimensions. Furthermore, the application of artificial intelligence and big data analytics represents an important direction for future research. Through processing and analyzing large-scale neural data, researchers can unveil hidden laws and patterns within the data, leading to a deeper understanding of the relationship between BNST and anxiety. By leveraging these technologies, smarter and more personalized treatment plans can be developed to effectively help patients with anxiety disorders.
